# A muramidase from *Acremonium alcalophilum* hydrolyse peptidoglycan found in the gastrointestinal tract of broiler chickens

**DOI:** 10.1093/jimb/kuab008

**Published:** 2021-02-04

**Authors:** Carsten Østergaard Frederiksen, Marianne Thorup Cohn, Lars Kobberøe Skov, Esben Gjerløff Wedebye Schmidt, Kirk Matthew Schnorr, Steen Buskov, Miika Leppänen, Ilari Maasilta, Estefania Perez-Calvo, Rual Lopez-Ulibarri, Mikkel Klausen

**Affiliations:** Novozymes A/S, Kalundborg, DK-4400, Denmark; Novozymes A/S, Lyngby, DK-2800, Denmark; Novozymes A/S, Lyngby, DK-2800, Denmark; Novozymes A/S, Bagsværd, DK-2880, Denmark; Novozymes A/S, Lyngby, DK-2800, Denmark; Novozymes A/S, Kalundborg, DK-4400, Denmark; Department of Biological and Environmental Sciences and Department of Physics, University of Jyvaskyla, Jyvaskyla, FI-40014, Finland; Department of Physics, University of Jyvaskyla, Jyvaskyla, FI-40014, Finland; Research Centre for Animal Nutrition and Health, DSM Nutritional Products, Village-Neuf, F-68305 Saint Louis, France; DSM Nutritional Products AG, Basel, Switzerland; Novozymes A/S, Kalundborg, DK-4400, Denmark

**Keywords:** Muramidase, Feed additive, Chickens

## Abstract

This study evaluates peptidoglycan hydrolysis by a microbial muramidase from the fungus *Acremonium alcalophilum in vitro* and in the gastrointestinal tract of broiler chickens. Peptidoglycan used for i*n vitro* studies was derived from 5 gram-positive chicken gut isolate type strains. *In vitro* peptidoglycan hydrolysis was studied by three approaches: (a) helium ion microscopy to identify visual phenotypes of hydrolysis, (b) reducing end assay to quantify solubilization of peptidoglycan fragments, and (c) mass spectroscopy to estimate relative abundances of soluble substrates and reaction products. Visual effects of peptidoglycan hydrolysis could be observed by helium ion microscopy and the increase in abundance of soluble peptidoglycan due to hydrolysis was quantified by a reducing end assay. Mass spectroscopy confirmed the release of hydrolysis products and identified muropeptides from the five different peptidoglycan sources. Peptidoglycan hydrolysis in chicken crop, jejunum, and caecum samples was measured by quantifying the total and soluble muramic acid content. A significant increase in the proportion of the soluble muramic acid was observed in all three segments upon inclusion of the microbial muramidase in the diet.

## Introduction

The gastrointestinal tract of animals is home to a complex microbial ecosystem that live in close interaction with host cells. Bacteria in the gastrointestinal tract can be classified into three groups based on metabolic activity: (a) viable and active cells, (b) viable and inactive cells, and (c) dead cells. Attempts to quantify metabolic status of bacteria in the gastrointestinal tract have been reported for humans (faeces), Syrian hamsters (caeca), Arctic ground squirrels (caeca), and Rex rabbits (stomach, jejunum, ileum, colon, caecum) using flow cytometry and live/dead PCR. Two studies of human faecal samples contained on average 49 and 56% live cells, 19 and 27% injured cells, and 17 and 32% dead cells (Ben-Amor et al., 2005; Maurice et al., 2013). Caeca samples from Syrian hamsters and Arctic ground squirrels contained between 72 and 81% intact cells, 4 and 9% damaged cells, and 10 and 20% dead cells (Hatton et al., 2017; Sonoyama et al., 2009; Stevenson et al., 2014) Another study used a live/dead PCR approach to measure live and dead cells in Rex rabbits, and noted that 1–3% live cells were found in the foregut (stomach, jejunum, ileum), 25% in the caecum, and 19% in the colon. Injured cells are not quantified by live/dead PCR (Fu et al., 2018).

Bacterial community structure analysis is often done by DNA sequence analysis tools that cannot distinguish between the metabolic activity of bacteria. This leaves many questions about the impact of the bacteria in the three metabolic activity groups on animal physiology unanswered (Maurice et al., 2013). Live bacteria and injured bacteria use ingested nutrients for internal biological processes and produce output that can impact host physiology (Gentile & Weir, 2018). Dead bacteria can interact with the host by release of cell bound material that is normally contained inside live cells. Well-known examples are lipopolysaccharide (endotoxin), lipopeptides, peptidoglycan, lipoteichoic acid, double stranded RNA, and unmethylated DNA fragments (Beutler & Rietschel, 2003; Reith & Mayer, 2011).

Hydrolysis of specific cell debris components is one way of investigating cell debris interaction with the host. Supplementation of the peptidoglycan hydrolysing microbial muramidase from *Acremonium alcalophilum* expressed in *Trichoderma reesei* have been reported to increase body weight and decrease feed conversion ratios of broiler chickens without reducing host microflora counts of enterobacteria and *Lactobacillus* (Goodarzi Boroojeni et al., 2019; Lichtenberg et al., 2017; Sais et al., 2019). *In vivo* effects of supplementing other peptidoglycan hydrolysing enzymes; hen egg white lysozyme (Long et al., 2016; Nyachoti et al., 2012; Oliver & Wells, 2015) and human lysozyme has been reported in literature as well (Cooper et al., 2014; Maga et al., 2012). Physiological effects of neutralizing small circulating peptidoglycan fragment with antibodies in mice have also been described (Huang et al., 2019). 

Peptidoglycan is a key structural component of bacterial cell walls, positioned on the outside of the bacterial cytoplasmic membrane. Its key function in live cells is to keep cell integrity by withstanding osmotic pressure (turgor). Peptidoglycan also functions to give cell shape (e.g., sphere, rod, and spiral) and as an anchor point for cell envelope components, such as proteins and teichoic acids (Vollmer et al., 2008a). Peptidoglycan is composed of polymers of alternating β-(1→4)-linked *N*-acetyl-glycosamine (Glc*N*Ac) and *N*-acetyl-muramic acid (Mur*N*Ac) sugar residues. The Glc*N*Ac Mur*N*Ac polymers are crosslinked by short peptides which differ in composition between species. Key unique features of peptidoglycan include muramic acid d-amino acids and unusual amide bonds (Vollmer & Seligman, 2010).

Muramidases also known as lysozymes or *N*-acetylmuramidases have been grouped into different families based on sequence and 3D structure: Hen egg white lysozyme (GH22 family), goose egg white lysozyme (GH23 family), bacteriophage T4 lysozyme (GH24 family), Sphingomonas flagellar protein (GH73 family), and Chalaropsis lysozymes (GH25 family) are the most well-known examples from each family (Korczynska et al., 2010). The *A. alcalophilum* muramidase described in this paper belong to the GH25 family (Lichtenberg et al., 2017).

A number of different effects of enzymatic peptidoglycan hydrolysis have been reported in the literature. One is the antimicrobial effect on live bacteria, first discovered by Alexander Flemming in 1922. This effect has been described for human, hen egg white lysozymes and others (Jollès & Jollès, 1984). Peptidoglycan hydrolysing enzymes without antimicrobial potency are especially important for bacterial physiology as they are involved in regulating cell wall turnover during growth as well as separation of cells during cell division (Vollmer et al., 2008b). Peptidoglycan hydrolysis has also been mentioned to impact immune function through enhancing or dampening the immune response. This is believed to happen through removal of polymeric peptidoglycan and/or production of small bioactive peptidoglycan fragments such as muramyl dipeptide (Inohara et al., 2003; Ragland & Criss, 2017). High and low molecular weight soluble peptidoglycan prepared by enzyme hydrolysis or sonication have been found to initiate different biological responses when delivered intravenously to rats (Chetty et al., 1982; Fox et al., 1982). The metabolic fate of small ^14^C labelled peptidoglycan fragments have also been studied in animal models. This includes muramyl di-, tri-, and pentapeptides. When delivered intravenously or subcutaneously the major part of the muramyl peptides have been found to be excreted in the urine either intact or as the corresponding peptide. When delivered orally muramyl peptides are retained in the intestinal tract for several hours after which they are to a large extent exhaled as CO_2_. This indicates that muramyl peptides can be degraded in the gastrointestinal tract (Ambler & Hudson, 1984; Valinger et al., 1987).

Because muramic acid is only found in peptidoglycan it has been used as a biomarker of microbial biomass in complex biological systems. In soil science, it has been used to determine microbial contribution to soil organic matter (Joergensen, 2018). In animal science, it has been used in one case to measure microbial biomass in faeces of dairy cows (Jost et al., 2013) and in several cases to track peptidoglycan in animal tissue (Fox et al., 1980).

Here, we report peptidoglycan hydrolysis efficacy of a fungal muramidase from *A. acremonium* to peptidoglycan preparations from *Enterococus gallinarum, Lactobacillus aviaries* subsp. *araffinosus, Lactobacillus kitasatonis, Bifidobacterium gallinarum*, and *Lactobacillus gallinarum*. All five strains are type strains, isolated from the gastrointestinal tract of chickens. They thus are contributing to the extracellular peptidoglycan released into the gastrointestinal tract upon bacterial cell division and death. It is thus likely that changes in animal physiology due to muramidase supplementation will be a result of hydrolysis of this kind of peptidoglycan. In addition, we report how quantification of total muramic acid content and soluble muramic content of intestinal samples can be used to measure the muramidase hydrolysis efficiency in the gastrointestinal tract.

## Materials and Methods

### Enzyme and Peptidoglycan

The *A. alcalophilum* (strain CBS 114.92) muramidase (GenBank ID: MN603156.1) was heterologously expressed in *T. reesei* as described previously (Lichtenberg et al., 2017). The fermentation supernatant was filtered through a Fast PES Bottle top filter with a 0.22 μm cut-off, and the pH was adjusted to 4.5 with 10% acetic acid. After the pH-adjustment the solution became slightly cloudy and was clarified by filtration through a Fast PES Bottle top filter with a 0.22 μm cut-off. Following this pre-treatment approximately 650 ml batches of the muramidase-containing solution were purified by cation-exchange chromatography on SP Sepharose, of an approximately 50 ml volume packed in a XK26 column, using 50 mM Na-acetate pH 4.5 as buffer A and 50 mM Na-acetate plus 1 M NaCl pH 4.5 as buffer B. The muramidase eluted from the column at approx. 0.3 M NaCl and fractions were pooled based on the chromatogram (absorption at 280 and 254 nm) and SDS–PAGE analysis. The pooled fractions were buffer-exchanged into 50 mM Na-acetate, pH 5.5 and concentrated using Amicon spin filters with a 10 kDa cut-off. The molecular weight, as estimated from SDS–PAGE, was approximately 22 kDa and the purity of both was >95%. Intact molecule mass spectrometry confirmed the expected protein molecular weight. The tested muramidase for chicken broiler diet supplementation was included as dry commercial formulated form. It had an analysed muramidase activity of 127 000 LSU(F)/g product analysed as previously described (Lichtenberg et al., 2017). The muramidase product is commercially available from DSM Nutritional Products, Kaiseraugst, Switzerland.

Purified peptidoglycan from *E. gallinarum* DSM24841^T^, *L. aviaries* subsp. *araffinosus* DSM 20653^T^, *L. kitasatonis* DSM 16761^T^, *B. gallinarum* DSM 20670^T^, and *L. gallinarum* DSM 10532^T^ was bought from the Leibniz Institute, DSMZ-German Collection of Microorganisms and Cell Cultures GmbH, Braunschweig, Germany. Samples were purified and characterized by thin-layer chromatography by DSMZ using standard protocols (Schumann, 2011).

### Peptidoglycan Hydrolysis

Peptidoglycan suspensions were prepared by mixing peptidoglycan samples with sterile physiological saltwater (0.9% NaCl) to a concentration of 6 mg/ml. Suspensions were stored overnight at 5°C. Samples were sonicated at 30 kHz (ultrasonic bath, Branson 5800) for 3 × 5 min and mixed thoroughly on a whirlymixer before pipetting. One hundred microlitres of each peptidoglycan stock (6 mg/ml) was then mixed with 800 μl citric acid—phosphate buffer pH 6 and 100 μl muramidase solution (or dilution buffer in blank samples). End concentration in tubes equalled 25 μg/ml muramidase and 0.6 mg/ml peptidoglycan. Hydrolysis was performed for 2 hr, 40°C, 300 rpm in a thermomixer and enzyme reaction was stopped by incubation at 95°C for 15 min.

### Reducing End Peptidoglycan Solubilization Assay

Purified peptidoglycan preparations are insoluble, as part of the purification step involves several washing steps (Schumann, 2011). Peptidoglycan enzyme hydrolysis is used in the literature to increase the pool of soluble fragments, which was used to measure peptidoglycan hydrolysis efficacy (Anderson et al., 2019; van der Aart et al., 2018). Here, we used a reducing end assay to quantify the amounts of reducing sugars in an acid hydrolysed supernatant. In order to quantify peptidoglycan solubilization by this approach 500 μl of control and hydrolysed peptidoglycan samples were centrifuged at 4,000 *g* for 5 min. One hundred microlitres of the supernatant was mixed with 50 μl of 3.2 M HCl and incubated for 80 min at 95°C. The pH was increased by addition of 50 μl 3.5 M NaOH. A 150 μl aliquot was mixed with 75 μl of 4-hydroxybenzoic acid hydrazide (PAHBAH) and incubated for 10 min at 95°C after which absorbance of a 150 μl sample was measured at 405 nm using a microtiter plate reader.

### Helium Ion Microscopy

Both intact and hydrolysed peptidoglycan were imaged with a helium ion microscope (Zeiss Orion Nanofab, Nanoscience Center, Univeristy of Jyväskylä), following procedures described previously (Leppänen et al., 2017). Silicon substrates were washed with 70% EtOH and milliQ water. After that, substrates were hydrophilized with SC7620 (Quorum Technologies Ltd) glow discharge unit and incubated in the poly-l-lysine (0.01%, Mol. Wt. 150k-300k, Sigma-Aldrich) for 10 min. Then substrates were washed 3 times with sterile milliQ water and left to dry in the air until usage (within one week after the preparation). Peptidoglycan samples were thawed at 4°C, diluted 1 : 10 in sterile water and pipetted over the prepared substrate in a 48 well plate and allowed to adhere at 4°C on an agitator for 4 h. After immobilization, samples were fixed with a 2% glutaraldehyde fixative in 0.1 M sodium cacodylate (NaCac) buffer for 4 h and washed two times for 5 min with 0.1 M NaCac, osmicated 30 min in 1% OsO_4_ in NaCac, washed three times for 5 min with 0.1 M NaCac and dehydrated with ethanol in a step series of 30, 50, 70, 90%, and 2 times with 99%, 15 min–1 h each. After dehydration, ethanol was replaced with 1 : 1 hexamethyldisilazane (HDMS):ethanol for 5 min and then 100% HMDS for 5 min and finally excess HMDS was pipetted out and samples were left to evaporate overnight. All of the buffers used were prepared to pH 7. Imaging was done using an acceleration voltage of 30 kV, aperture of 10 μm and a spot size of 6 or 7 resulting in an ion current of 0.2–0.4 pA. Charge compensation with the electron flood gun was used with some samples to prevent charging.

### Mass Spectroscopy of Intact and Hydrolysed Peptidoglycan

Samples (20 mg freeze-dried or 100 μl extract) were transferred to Eppendorf tubes. Ninety microlitres water and 10 μl 4 M NaOH was added and mixed thoroughly. One hundred microlitres 0.5 M PMP solution (1-phenyl-3-methyl-5-pyrazolone) in MeOH was added followed by mixing and incubation at 70°C for 30 min. Samples were cooled before addition of 10 μl 4 M HCl. Two hundred microlitres MeOH was added followed by mixing and centrifugation (5 min at 13 000 *g*). The supernatants were transferred to HPLC-vials and analysed by exact mass LCMS.

Analysis for PMP-muropeptides was done on using an Accela UPLC with HTC autosampler. Detection was done using a Q Exactive mass spectrometer (Thermo Scientific). The separation was done on a CSH C18 column (150 × 2.1 mm, 1.7 μm, Waters) using gradient elution. Mobile phase A was 0.1% formic acid. Mobile phase B was 0.1% formic acid in acetonitrile. The gradient was initially 83% A which was held constant till 1 min, then increased linearly to 80% A in 9 min, the to 5% A in 0.50 min. At 11 min, the mobile phase composition was returned to initial conditions. Injection volume was 10 μl, column temperature 60°C and flow rate 500 μl/min.

The Q Exactive mass spectrometer was operated at the following conditions: Spray voltage: 3.5 kV, sheath gas flow: 50, aux gas flow: 10, sweep gas flow: 3, capillary temperature: 250°C, aux gas heater: 350°C. Scan range was 150–1,800 amu, resolution set to 70 000, AGC target 3E6 and maximum IT was 200 ms.

Datafiles were processed in Refiner (Genedata) with identification of muropeptides using an inhouse database. The summed intensity of identified peaks was used for further data evaluation. Signal from identical annotated muropeptides were summed and log2 transformed. Missing values were set to 1.

### Silkworm Larvae Plasma Assay

Peptidoglycan activation of the prophenoloxidase cascade in silkworm larvae plasma (SLP) has been used to quantify peptidoglycan (Tsuchiya et al., 1996). Control and hydrolysed peptidoglycan suspensions, were measured using the SLP Multi-Test Reagent Set from Wako Pure Chemical Industries, Ltd., Osaka, Japan. Assays was performed in 96 well microtiter plates as described in the test set protocol. Briefly 50 μl peptidoglycan suspensions were diluted 30 times in physiological 0.9% salt water mixed with 50 μl SLP test solution and incubated at 30°C for 60 min. Absorbance development was measured at 650 nm as a measure of prophenoloxidase cascade activation.

### Collection of Broiler Digesta Samples

An *in vivo* study with broiler chickens was conducted at the DSM Research Center of Animal Nutrition in Village Neuf (France) according to the to the official French guidelines for the protection of animals used for scientific purposes and conformed to the European Union Guidelines (Directive 2010/63/EU). A total of 576 male 1-day-old Cobb 500 broilers were obtained from a local hatchery and randomly divided into 32 pens (18 chickens per pen) on wood shavings. Water and a maize–wheat– soybean-based diet, formulated to be nutritionally adequate according to NRC (1994), were provided *ad libitum*. Randomly selected pens were allocated to one of two experimental treatments: a control diet (CTR) or CTR diet supplemented with the microbial muramidase from *A. alcalophilum* at 45 000 LSU(F)/kg feed, 16 pens each. The light schedule was an 18-h light/6-h dark cycle. Infrared bulbs (1 per pen during the first week) together with a central heating system provided the optimal temperature. Birds remained healthy throughout the study and the growth performance parameters were in line with the reference values of Cobb 500 at the same age.

At 36 days of age, one chicken per pen (16 chickens per treatment) were euthanized and the content from crop, jejunum, and caecum were collected. Samples were snap frozen in liquid nitrogen and stored at −20°C for determination of muramic acid concentration.

### Muramic Acid Peptidoglycan Hydrolysis Assay Applied to Broiler Digesta Samples

Digesta samples were freeze dried and grinded to ensure homogeneity and 100 mg of each sample were collected to determine the amount of muramic acid in total dry matter. Another 100 mg were mixed with 0.8 ml citric acid—phosphate buffer at pH 6 and incubated 95°C for 15 min, to inactivate muramidase activity. Samples were then extracted for 45 min at 23°C with shaking after which they were centrifuged at 13 000 RPM at 5°C for 5 min. Supernatants were then decanted into new tubes. Solid and liquid samples was transferred to GC vials and hydrolysed using an end concentration of 5 M hydrochloric acid for 24 hr at 100°C. The hydrolysate was then dried in a freeze dryer under vacuum. The dried hydrolysate was reconstituted in ultrapure water and centrifuged. Two hundred microlitres supernatant was transferred to an HPLC vial and 20 μl 4 M NaOH, 20 μl 0.1 mg/ml 6-deoxy-d-glucose (internal standard), and 200 μl 0.5 M PMP solution (1-phenyl-3-methyl-5-pyrazolone) in MeOH was added followed by mixing and incubation at 70°C for 30 min. Samples were cooled before addition of 20 μl 4 M HCl and 400 μl MeOH followed by mixing and centrifugation (5 min at 13 000 *g*). The supernatant was diluted in 50% methanol and transferred to HPLC vials and analysed by LCMS. Determination of muramic acid was done using an Acquity UPLC (Waters). Detection was done using a Xevo TQ-S micro tandem quadrupole mass spectrometer (Waters). The separation was done on an Acquity UPLC CSH C18 column (50 × 2.1 mm, 1.7 μm, Waters) using gradient elution. Mobile phase A was 0.1% formic acid. Mobile phase B was 0.1% formic acid in acetonitrile. The initial condition of 90% A was held constant until 1 min, then increased linearly to 80% A in 9 min, then to 5% A in 0.1 min. At 11 min, the mobile phase composition was returned to initial conditions. Injection volume was 5 μl, column temperature 60°C and flow rate 500 μl/min. The Xevo TQ-S micro mass spectrometer was operated at the following conditions: Capillary voltage: 3 kV, cone voltage: 20 V, source temperature: 150°C, desolvation temperature: 500°C, desolvation gas flow 600 l/hr, cone gas flow: 20 l/hr. Datafiles were processed using TargetLynx software (MassLynx, Waters).

The amount of collected digesta samples were in some cases below the amount needed for analysis. 12 crop samples, 11 jejunum samples, and 11 caeca samples in the control group and 9 crop samples, 11 jejunum, and 12 caeca samples were analysed in the muramidase group.

### Statistic Processing of Data

JMP^®^, Version 14.1.0 SAS Institute Inc., Cary, NC, 1989–2019 was used for *t*-test of paired means for reducing ends analysis and SLP data to test for significant differences between peptidoglycan samples and hydrolysed peptidoglycan samples. XLSTAT statistical and data analysis solution, Version 2019.1.2 Addinsoft Boston, USA, was used to identify putative muropeptides with significantly different abundance by use of a parametric test with Benjamini–Hochberg multiple correction.

## Results and Discussion

### Microscopic Characterization of Isolated Peptidoglycan Before and After Hydrolysis

**Fig. 1.**
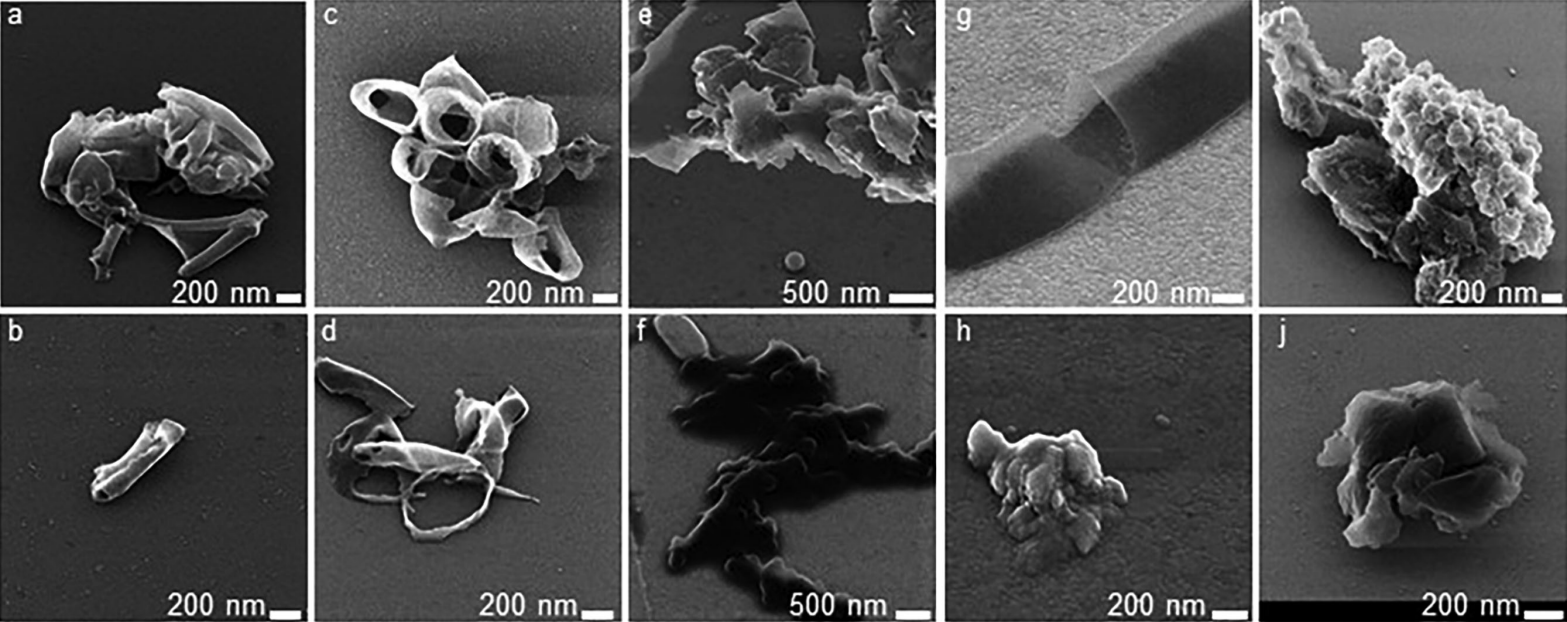
Helium ion microscopy images of peptidoglycan and hydrolysed peptidoglycan. Top row pictures show peptidoglycan and bottom row pictures show hydrolysed peptidoglycan, respectively, of *B. gallinarum* (a and b), *E. gallinarum* (c and d), *L. aviaries* (e and f), *L. gallinarum* (g and h), and *L. kitasatonis* (i and j).

Visual qualitative effects of muramidase peptidoglycan hydrolysis were investigated by helium ion microscopy for the first time. This approach aimed at characterizing the visual phenotype of the purified peptidoglycan and to test whether hydrolysis would change the appearance of the samples. Representative micrographs are shown in Fig. 1. Bacterial rod and cocci shapes were recognizable, respectively, for *L. gallinarum* and *E. gallinarum* preparations– whereas peptidoglycan from the other three preparations were fragmented to a level where cocci or rod shapes were not visible. Debris structures were less frequently found in hydrolysed samples compared to control peptidoglycan samples. The data thus support that tested muramidase can hydrolyse peptidoglycan, found in the gastrointestinal tract of chickens. Morphology of bacterial cell wall fragments has previously been studied by optical super-resolution microscopy (Turner et al., 2018; Verwer & Nanninga, 1976; Wheeler et al., 2011), atomic force microscopy (Turner et al., 2018; Verwer & Nanninga, 1976; Wheeler et al., 2011), and transmission electron microscopy (Verwer & Nanninga, 1976). The effect of hen egg white lysozyme on cell morphology has been studied on intact cells of *Bacillus subtilis* (Tulum et al., 2019).

### Solubilization of Peptidoglycan Quantified by Acid Hydrolysis by Reducing End Assay

**Fig. 2.**
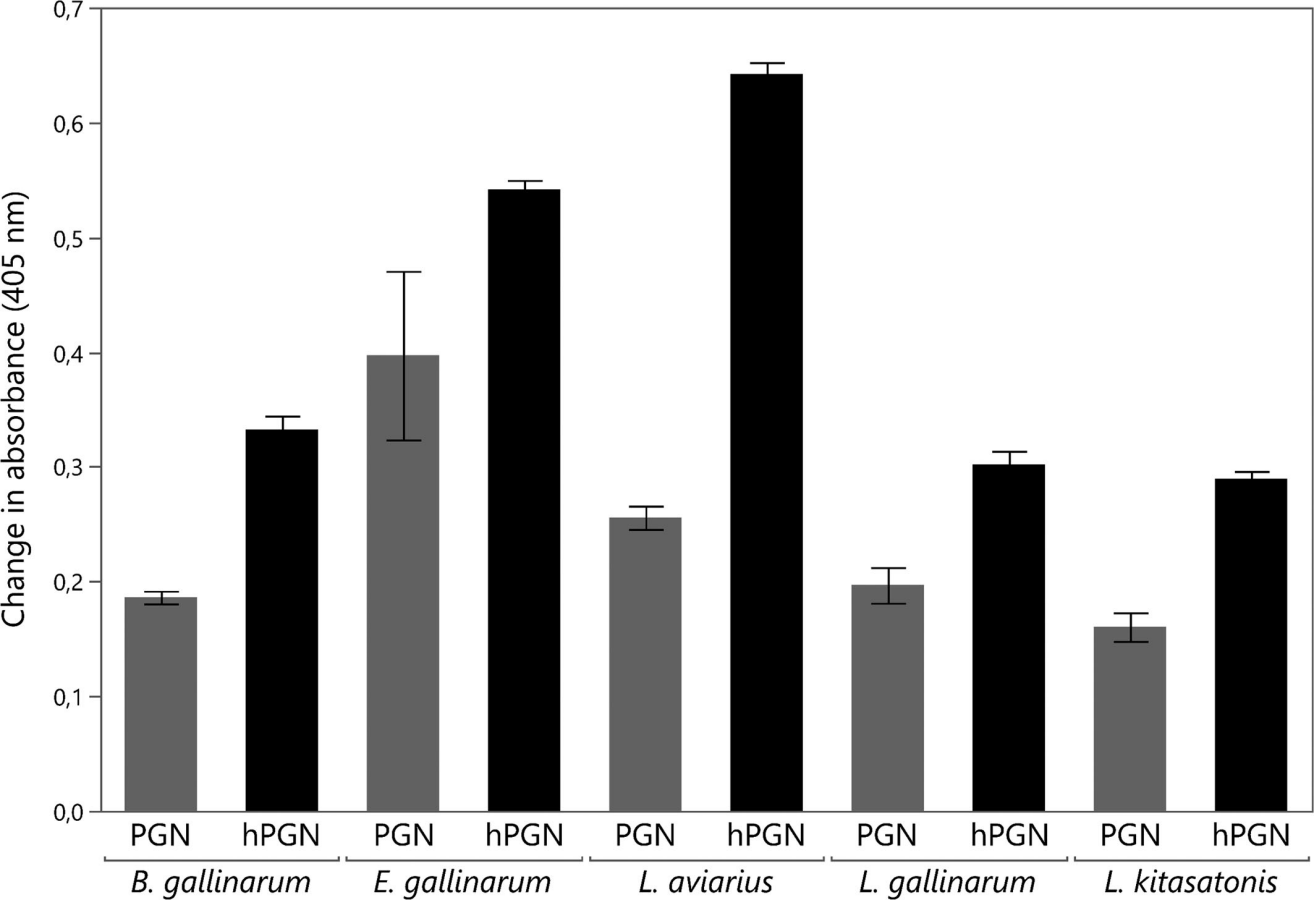
Changes in absorbance (405 nm) reflecting the amount of soluble peptidoglycan in control (PGN) and hydrolysed peptidoglycan (hPGN) from *B. gallinarum, E. gallinarum, L. aviarius, L. gallinarium*, and *L. kitasatonis*. Mean values and standard deviations were calculated from triplicates. The increase in means due to muramidase hydrolysis was found to be significant across the five samples using a paired sample *t*-test (*p* < .001) comparing control peptidoglycan with hydrolysed peptidoglycan.

Reducing sugars of acid hydrolysed muramidase reaction supernatants were quantified to estimate peptidoglycan solubilization as a result of hydrolysis, This approach would complement the microscopy data to demonstrate peptidoglycan hydrolysis by the muramidase. As seen in Fig. 2, all five preparations of control peptidoglycan already contained measurable numbers of soluble peptidoglycan, probably due to the mechanical breakdown of peptidoglycan during the sampling procedure. *B. gallinarum, L. gallinarum*, and *L. kitasotonis* all had the lowest concentration of soluble peptidoglycan giving a response close to 0.2 absorbance units. *L. aviaries* gave a slightly higher response of 0.25 absorbance units and *E. gallinarium* gave the highest response at 0.40. The amount of soluble peptidoglycan numerically increased for all five peptidoglycan preparations upon muramidase supplementation. The highest increase in the number of reducing ends was observed for *L. aviarius* whereas the other four peptidoglycans increased by a similar value. A paired *t*-test was used to test if peptidoglycan incubation with muramidase significantly increased the concentration of soluble peptidoglycan across the five samples. The mean increase of 0.18 absorbance units (A405 nm) in hydrolysed peptidoglycan samples compared to control peptidoglycan samples not being subjected to the muramidase was found to be significant (*p* < .0001). Measurement of reducing sugars is a common method to measure enzyme activity of enzymes such as carbohydrases, since reducing sugars are formed as result of the enzymatic hydrolysis between two carbohydrates (Gusakov et al., 2011). Reducing sugar-based assays have previously been reported used as a tool to study substrate specificity of the *N*,6-*O*-diacetylmuramidase from *Streptomyces globisporus* (Seo et al., 2003) Other methods to study peptidoglycan hydrolysis includes fluorescein isothiocyanate or Remazol Brilliant Blue labelled peptidoglycan-based assays or turbidity reduction assays (Maeda, 1980; Santin & Cascales, 2017). A turbidity-based study of hen egg white lysozyme, λ lysozyme, mutanolysin, T4 lysozyme, goose egg white lysozyme, and cauliflower lysozyme hydrolysis of peptidoglycan from *Micrococcus luteus, Salmonella typhimurium, Escherichia coli, Yersisnia enterocolitica*, and *Pseudomonas auruginosa*, showed differences in efficacy dependent on enzyme substrate combinations even between closely related bacteria (Nakimbugwe et al., 2006). The reason behind the observed differences was not described. This current paper also observed efficacy differences between the closely related *Lactobacillus* samples. Secondary glycan strand structural variations such as *N*-deacylation, *N*-glycolylation, and *O*-acetylation in the glycan strands could be one reason for the observed differences, as these are known to be dynamic and can change with the physiological state of the cell (Vollmer, 2008). The reducing end data confirms the microscopy data show activity of the muramidase on all tested substrates.

### Mass Spectroscopy of Intact and Hydrolysed Peptidoglycan

**Fig. 3.**
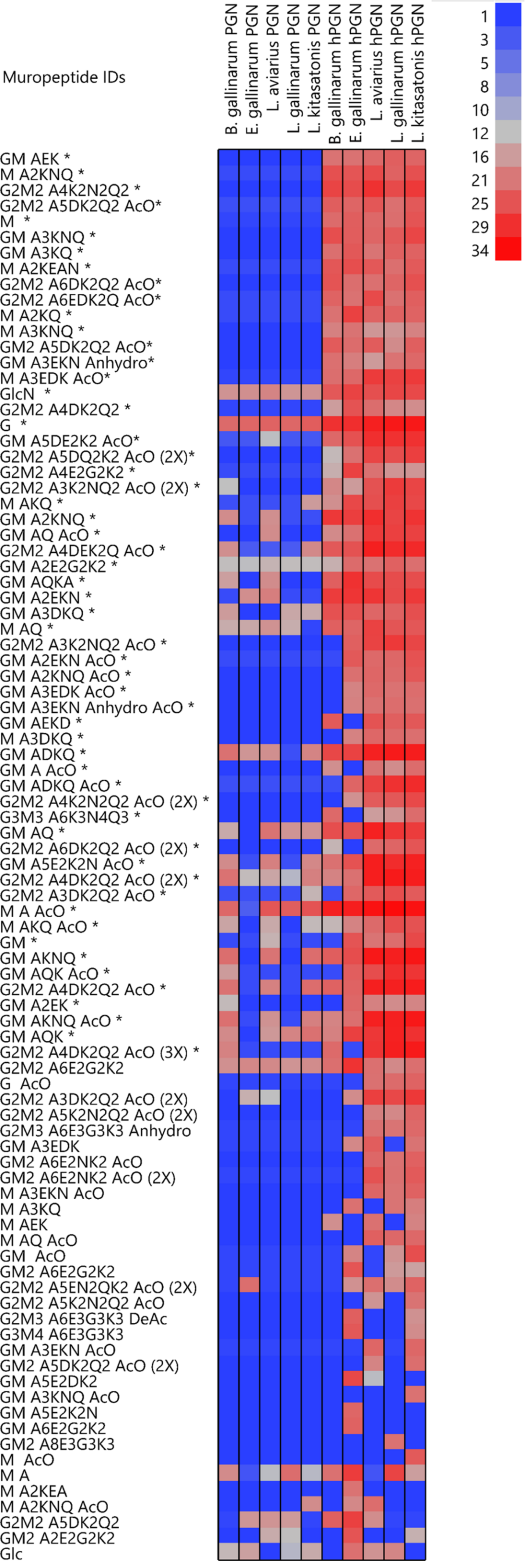
Mass spectroscopy comparison of muropeptide relative abundance from peptidoglycan (PGN) and hydrolysed peptidoglycan (hPGN) in *B. gallinarum, E. gallinarum, L. aviarius, L. gallinarium*, and *L. kitasatonis* and muramidase. Heatmap shows values of log2 transformed intensity scores of each muropeptide. Muropeptide composition is listed on the *x*-axis using the following abbreviation: M = *N*-acetylmuramic acid and G = *N*-acetylglycosamine for glycan chain; A = alanine; E = glutamic acid; G = glycine; K = lysine; N = asparagine; Q = glutamine for peptide bridge; and AcO = *O*-acetylation, DeAc = *N*-deacetylation, and Anhydro = 1,6-anhydro-MurNAc for glycan chain modifications. Muropeptides are sorted according to *p* values from smallest to largest, muropeptide IDs with an asterisk indicate the signal from muropeptide is significantly higher in hPGN samples (*n* = 5) compared to PGN samples (*n* = 5) (*p* level .05 parametric test with Benjamini–Hochberg multiple correction).

Mass spectroscopy was used to study peptidoglycan hydrolysis by comparing the relative abundance of reaction products to control peptidoglycan (Fig. 3). Ninety putative peptidoglycan fragments were identified. As microscopy and the reducing end assay focus on changes in the substrate, mass spectroscopy quantifies production of hydrolysis reaction products. Fragments with different combinations of *N*-acetylglucosamine and *N*-acetylmuramic acid, peptide bridge amino acids and glycan chain modifications *O*-glycolylation, de-*N*-acetylation, and 1,6-anhydroMur*N*Ac. Eight different combinations of *N*-acetylglucosamine and *N*-acetylmuramic acid were identified. Thirty-seven putative peptide bridge structures bound to glycan fragments were identified. Peptide bridges were made up of seven different amino acids. Glucose and d-glucosamine sugars were also identified. Three modifications of the glycan chains were identified. Log2 mass spectroscopy signal values across the five different control peptidoglycan samples were compared to muramidase hydrolysed samples. Fifty-eight out of 90 of the identified hydrolysis products were significantly higher in abundance after hydrolysis compared to before hydrolysis.

Mass spectroscopy has previously been used for the purpose of elucidating peptidoglycan structure and typically includes enzyme digestion prior to analysis to solubilize the fragments. Eight-three muropeptides were identified following hydrolysis of *Clostridium difficile* peptidoglycan with Mutanolysin, 80 different muropeptides from *Streptomyces coelicolor* were identified following hydrolysis by Mutanolysin and hen egg white lysozyme and 160 muropeptides were identified in a study of *Pseudomonas aeruginosa* biofilms following mutanolysis peptidoglycan hydrolysis (Anderson et al., 2019; Bern et al., 2017; van der Aart et al., 2018). Extraction of soluble peptidoglycan fragments using *Chalaropsis B* muramidase has also been described in the literature (Rosenthal & Dziarski, 1994). The mass spectroscopy data confirmed that the tested muramidase can hydrolyse all tested peptidoglycan preparations as was also shown by microscopy and the reducing end assay.

### Silk Larvae Plasma Assay

**Fig. 4.**
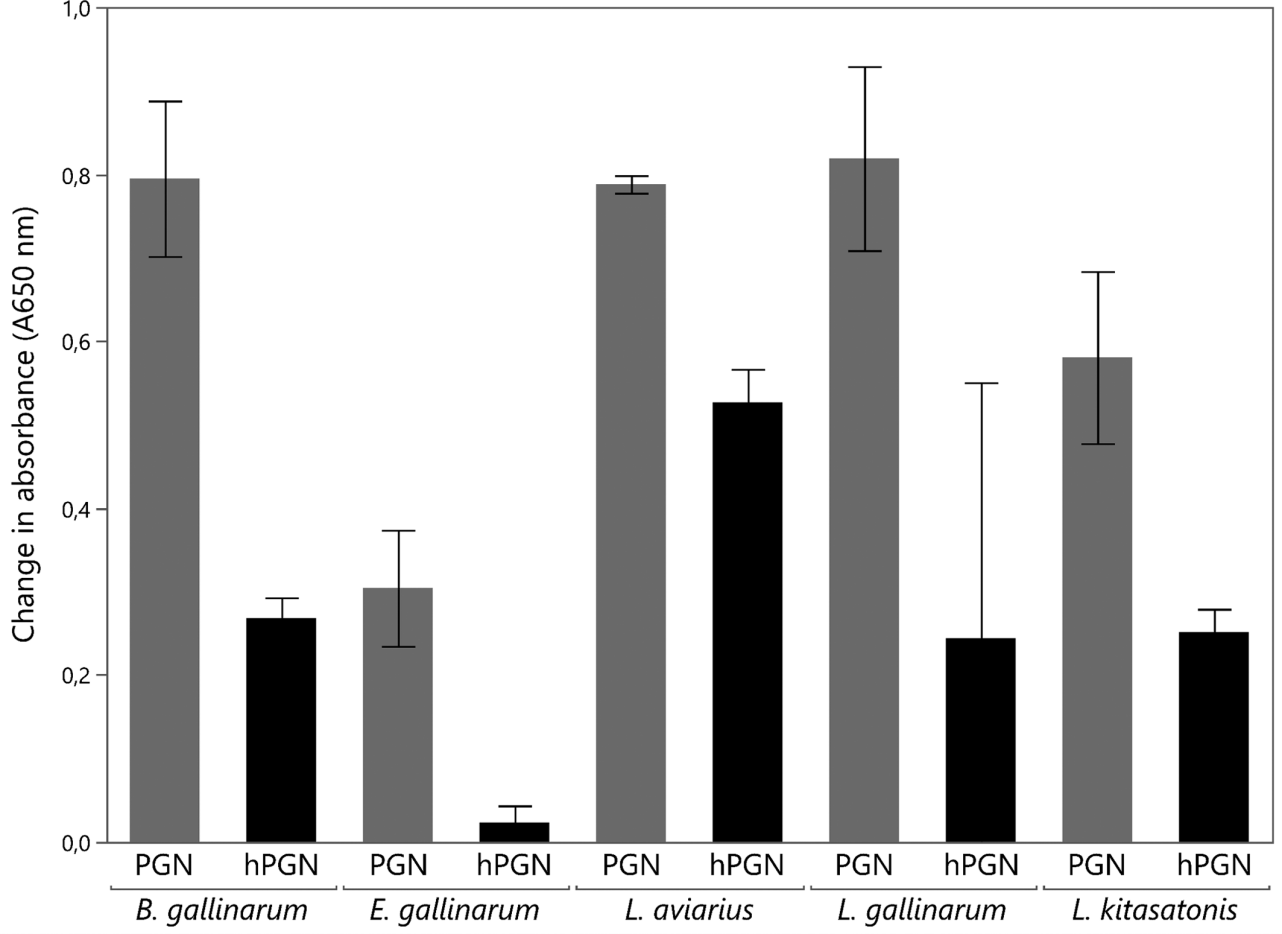
Changes in absorbance (650 nm) reflecting the amount of peptidoglycan recognition protein bound peptidoglycan (PGN) and hydrolysed peptidoglycan (hPGN) from *B. gallinarum, E. gallinarum, L. aviarius, L. gallinarium*, and *L. kitasatonis* (grey). Mean values and standard deviations were calculated from duplicates. The decrease in means due to muramidase hydrolysis was found to be significant across all samples using paired sample *t*-test *(p* < .001).

Peptidoglycan activates the prophenoloxidase cascade in the hemolymph of the silkworm, *Bombyx mori* (Tsuchiya et al., 1996). SLP can thus be used to test if control peptidoglycan and muramidase hydrolysed peptidoglycan, activates the prophenoloxidase cascade to different degrees. This would be an indication of how much muramidase hydrolysis changes the biological properties of the tested peptidoglycan. All five preparations of peptidoglycan activated the prophenol cascade prior to hydrolysis as seen in Fig. 4. *B. gallinarum, L. aviaries*, and *L. gallinarum* were activated the most. *L. kitasotonis* activated the cascade to a smaller degree and *E. gallinarum* activated the least. Muramidase hydrolysed peptidoglycan from all five bacteria activated the prophenol cascade as well, but to a smaller degree than the control samples. Hydrolysed peptidoglycan from *E. gallinarum* almost did not activate the pathway, whereas hydrolysed peptidoglycan from *B. gallinarum, L. gallinarum*, and *L. kitasatonis* had their activation reduced to half level compared to before hydrolysis. Hydrolysed *L. aviarius* was activated 75% less compared to the intact peptidoglycan. A paired *t*-test was used across samples to test if muramidase peptidoglycan hydrolysis significantly decreased activation of the prophenol cascade. The mean decrease of 0.39 absorbance (A650 nm) units (A650 nm) in hydrolysed samples, compared to unhydrolysed samples was found to be significant (*p* < .0002).

Interaction of peptidoglycan fragments with host receptors has been a research field since 1970 where chemical synthesis was used to find the minimal structure that activates an immune response in animals. Muramyl dipeptide was identified as the minimal structure that is recognized (Kusumoto et al., 2010). Longer structures than muramyl dipeptides were later found to interact with receptors as well as in a model system where mono-, di-, tetra-, and octa saccharides were synthetized and tested for Nod2 dependent tumour necrosis factor-α induction in human monocytes (Inamura et al., 2006). The minimum structure of peptidoglycan from *M. luteus* that induced antimicrobial activity in *B. mori* has been estimated to be (GlcNAc-MurNAc)_2_ with MurNAc substituted side chains. Hen egg white lysozyme hydrolysis has previously been used to prepare peptidoglycan of different sizes (Iketani et al., 1999). No data has, to our knowledge, been reported that describes the study of the minimal peptidoglycan structure that activates the prophenol oxidase cascade in *B. mori*. However, our data support that the tested muramidase hydrolyses peptidoglycan effectively enough to impact recognition of the peptidoglycan by a peptidoglycan recognition protein used in the silk worm cascade.

### Quantification of Peptidoglycan Hydrolysis in Intestinal Samples

As muramic acid is only found in bacterial peptidoglycan, it was quantified as a biomarker for peptidoglycan in two fractions of gastrointestinal content. By measuring total muramic acid content of digesta samples, as well as soluble peptidoglycan in the supernatant of digesta sample slurries, it was possible to identify the effects of muramidase inclusion in broiler chicken diets (Table 1). Data from analysed digesta samples support that the tested muramidase is capable of hydrolysing peptidoglycan found in the gastrointestinal tract of chickens. The principle of measuring enzyme hydrolysis by quantifying solubilized reaction products is commonly used to measure the effect of carbohydrases in the gastrointestinal tract (Choct et al., 2004; Rosenthal & Dziarski, 1994). A numeric but non-significant reduction in total and soluble muramic acid was found in crop samples. A significant effect of muramidase supplementation was observed when calculating soluble muramic acid as a % of the total muramic acid for each sample analysed. Crop soluble muramic acid (% of total) significantly increased from 37 to 46% upon addition of muramidase.

**Table 1.** Muramic Acid Concentration in Digesta Dry Matter and Soluble Extractions of Digesta Dry Matter. Samples from Experimental Control and Muramidase Diet Group was Compared Within Each Segment

Intestinal segment	Experimental group	Muramic acid (mg/kg), soluble	SD	Muramic acid (mg/kg), total	SD	Muramic acid (soluble, % of total)	SD
Crop	Control	80	53	215	138	37^a^	5
	Muramidase	74	35	172	95	46^b^	7
Jejunum	Control	108	89	336	269	32^a^	6
	Muramidase	102	94	167	162	66^b^	14
Caecum	Control	346^a^	91	3,733	441	9^a^	2
	Muramidase	559^b^	164	3,464	336	16^b^	6

*Note*. Values with a different superscript letters within an intestinal segment are significantly different at *p* < .05 (Student's *t*-test). SD = standard deviation.

Soluble muramic acid content of jejunum samples numerically increased in muramidase supplemented broilers, whereas total muramic acid number decreased on average. Soluble muramic acid (% of total) significantly increased from 32 to 66% when comparing control samples with muramidase supplemented samples.

Concentration of soluble muramic acid in the caecum significantly increased from 346 mg/kg in the control group to 559 mg/kg in the muramidase group. A numeric non-significant decrease in total muramic acid was observed and a significant increase from 9 to 16% was observed when comparing soluble muramic acid (% of total) numbers. The amounts of total muramic acid in the caecum was 19 times higher compared to crop concentration levels and 14 times higher than concentrations found in the jejunum. This is in line with the higher number of bacteria reported in the caecum compared to the crop and ileum (Rehman et al., 2007). Numeric, but nonsignificant, decreases in total muramic acid could be explained by intestinal degradation of muramic acid as described in the literature (Valinger et al., 1987). Peptidoglycan hydrolysis has to our knowledge not been measured before by comparing muramic acid content in a slurry of digesta samples relative to muramic acid content in total dry content. Total muramic acid concentration in cow faecal samples were found to be 460 mg/kg dry weight whereas soil concentrations varied between 27 and 210 mg/kg dry weight (Joergensen, 2018). The relative concentration of soluble peptidoglycan was not measured in these studies. Muramic acid has also been measured in dust (concentration 15 ng/mg dust) from swine facilities (concentration 5 ng/mg dust) and Dairy Barns (Poole et al., 2010) as a measure of bacterial load in dust. Muramic acid has also served as a marker of peptidoglycan in mammalian tissue. Publications about the concentration of peptidoglycan in healthy tissue are somewhat contradictory as some report it is as present and others not. There seems to be consensus though that peptidoglycan can be detected in diseased tissue, for example in cerebrospinal fluids from patients with Pneumococcal meningitis (Kozar et al., 2000, 2002).

## Conclusions

In summary, this study provides insight into hydrolysis of peptidoglycan by a muramidase from *A. alcalophilum in vitro* and in the gastrointestinal tract of broiler chickens. Helium ion microscopy, measurements of solubilization by reducing sugars, and mass spectroscopy confirmed hydrolysis of peptidoglycan from *E. gallinarum, L. aviaries* subsp. *araffinosus, L. kitasatonis, B. gallinarum*, and *L. gallinarum*. All type strains has been isolated from the gastrointestinal tract of chickens. Quantification of muramic acid was used to measure peptidoglycan hydrolysis in digesta samples from the crop, jejunum and caecum. Muramic acid content of digesta samples and digesta sample slurry supernatants were measured by mass spectroscopy. Effects on supplementing the *A. acremonium* muramidase to chicken diets could be measured as a significant increase in percent soluble to total muramic acid in all segments. A significant increase in soluble muramic acid content of caecum samples was also identified. Further data are needed to better understand the connection between bacterial cell debris, peptidoglycan, and animal physiology.

## Data Availability

The datasets generated during and/or analysed during the current study are available from the corresponding author on reasonable request.
